# ^13^ C NMR Studies of the Redox Reactions of Autothiomalate and Bis(Thiomalato)Gold(I) With Selenocyanate in an Aqueous Solution

**DOI:** 10.1155/MBD.1994.513

**Published:** 1994

**Authors:** Avarhusein A. Isab, M. Naseem Akhtar, Abdul Rahman Al-Arfaj

**Affiliations:** 1 Department of Chemistry King Fahd University of Petroleum and Minerals Dharan 31261 Saudi Arabia


					13C NMR STUDIES OF THE REDOX REACTIONS
OF AUTOTHIOMALATE AND BIS(THIOMALATO)GOLD(I)
WITH SELENOCYANATE IN AN AQUEOUS SOLUTION
Avarhusein A. Isab, M. Naseem Akhtar and Abdul Rahman AI-Arfaj
Department of Chemistry, King Fahd University of Petroleum and Minerals,
Dharan 31261, Saudi Arabia
Avarhusein A. Isab, M. Naseem Akhtar and Abdul Rahman AI-Arfaj
Department of Chemistry
King Fahd University of Petroleum and Minerals
Dhahran 31261, Saudi Arabia
The interactions of SCN- with aurothiomalate (AuStm) in aqueous solution were studied by 13C
NMR spectroscopy. The AuStm remains polymerized in the presence of SCN-, however, SeCN-
binds to AuStm forming the NCSe-Au-Stm- complex. This complex initially disproportionates to
give Au(SeCN)2- and Au(Stm)2-. The Au(SeCN)2- specie eventually decomposesto give metallic
gold and metallic selenium (or selenium anion). The free tmS- which is released from AuStm with
excess SeCN- is oxidized to thiomalic disulfide (tmS2). The bis complex Au(Stm)2 was
generated in aqueous solution and then reacted with SeCN-. Since Au(I) is blocked by both sides,
SeCN- did not form tmS-Au-SeCN- species. Instead, it gave selenium metal and Au(CN)2 In
the present studies we have demonstrated that SeCN- reacts differently with mono- and bis-
(thiomalato)gold(I).
513

				

